# The distinctive signature of regulatory CD4 T cells committed in the human thymus

**DOI:** 10.3389/fimmu.2025.1553554

**Published:** 2025-03-26

**Authors:** Alexandre A. S. F. Raposo, Susana Paço, Miguel Ângelo-Dias, Pedro Rosmaninho, Afonso R. M. Almeida, Ana E. Sousa

**Affiliations:** ^1^ GIMM - Gulbenkian Institute for Molecular Medcine, Lisbon, Portugal; ^2^ Faculdade de Medicina, Universidade de Lisboa, Lisboa, Portugal

**Keywords:** human thymus, human T-cell development, CD4 T cells, regulatory T cells, FOXP3, RNA-seq

## Abstract

Thymically committed regulatory CD4 T cells (tTregs) are essential for immune homeostasis and self-tolerance. We established the human tTreg Expression Signature by comparing genome-wide transcriptomic profiles between tTregs and their conventional counterparts (tTconvs). We further exploited the high sequencing depth of our bulk RNA-seq data to identify a subset of 250 genes significantly expressed in human tTregs and with neglectable expression in tTconvs, defined as below the levels of expression of *IL2RA*, that we named thymic Treg “private” genes. Notably, pathways related to cell motility, inflammation, and T-cell effector specification were overrepresented within the tTreg private genes. We found that 163 of these genes were significantly less expressed in circulating naïve and memory Tregs when compared to peripheral data generated in parallel. This result suggested a higher activity for most of the “private” genes in the thymus when compared to the peripheral compartments. Altogether, we provide a unique resource to inform future studies, such as for improving annotation in single-cell and spatial transcriptional data, or help in designing human studies to validate putative biomarkers for thymically committed Tregs, a priority in the field.

## 1 Introduction

CD4 regulatory T cells (Tregs) develop as a devoted lineage in the thymus and can also be induced from conventional T cells (Tconvs) in the periphery (1). Thymically committed Tregs (tTregs) are thought to be enriched in self-reactive T-cell receptors (TCRs) and to feature reduced plasticity, maintaining their suppressive properties in pro-inflammatory environments (1, 2). They are, therefore, essential for self-tolerance and immune homeostasis, preventing or limiting the activation and function of other T cells and immune cell populations (1–3).

Treg specification in the thymus is thought to occur via at least two pathways, one mainly relying on FOXP3 and TCR signaling, and the other on IL2RA (CD25) expression and IL2/IL15/STAT5 signaling (4–6). FOXP3 is described as the master regulator of Treg development, at the top of a hierarchy of transcriptional events defining the identity and function of Tregs, the “Treg signature” (7). Identifying the Treg signature in the human thymus is crucial to reveal factors whose deregulation may play a role in the pathogenesis of immune disorders and that may be targeted by therapeutic strategies (3).

Most studies based on high-throughput sequencing and characterizing molecular mechanisms that define human Tregs have focused on peripheral samples, including both thymic-committed and peripheral-induced Tregs, given the lack of markers to sort-purify tTregs (1, 3). However, notwithstanding the relevant information provided by T-cell development studies exploring the potential of murine models, particularly with fate-mapping experiments (8), there are fundamental differences between mice and humans that influence early Treg commitment and subsequent development (1–3, 7). For example, human thymocytes express MHC class II, allowing effective T-T cell selection (9), and express the antigen-presenting CD1a protein during early T-cell development that may modulate TCR signaling (10). Moreover, contrasting with the murine thymus, a clear population of FOXP3-expressing double-positive thymocytes is found in the human thymic cortex (5, 11). Thus, despite conservation for major thymocyte subpopulations (12), tTreg development must be addressed specifically in humans.

Single-cell sequencing allows the profiling of heterogeneous, rare cell populations and their developmental dynamics (13). This technique has been employed in the characterization of human thymus organogenesis and early T-cell commitment and development (14, 15). The first studies linking single-cell data to tissue localization through spatial transcriptomics have recently been published (15, 16). However, single-cell technology cannot yield the sequencing depth achieved by bulk RNA-seq and does not warrant full coverage of the universe of transcripts (17). Moreover, the definition of the common transcriptional profile of a population relies on bulk RNAseq data on sorted cells, a resource fundamental to support the annotation of the clusters identified by single-cell transcriptomics and to facilitate biomarker research (18).

Taking advantage of bulk RNAseq and ATACseq data, we recently reported transcription factor networks governing the human tTreg signature (19). Here, we further explored the expression signature that distinguishes tTregs from mature tTconvs and identified a subset of 250 differentially expressed genes (DEGs) with neglectable expression in tTconvs, as defined by being below the *IL2RA* expression level. These so-called tTreg “private” genes were enriched in pathways related to cell motility, T-cell functional specification, and inflammatory responses. Importantly, 163 tTreg “private” genes were significantly less expressed in circulating naïve and memory Tregs, supporting their role in Treg development in the human thymus.

## 2 Methods

### 2.1 Human samples

Thymic samples were obtained during pediatric reconstructive cardiac surgery, using tissue that would be otherwise discarded (1 male and 2 female children, between 1 and 24 months of age); peripheral blood samples were obtained from blood donors (3 female adults, between 22 and 33 years of age), as it was not possible to obtain enough blood from children to perform the required cell sorting. No evidence of immunodeficiency or syndromic diseases was found in any of the individuals. All participants or their legal representatives provided written informed consent. The study was approved by the ethical boards of the Lisbon Academic Medical Center and of the Hospital de Santa Cruz, Carnaxide, Portugal.

### 2.2 Cell sorting and flow cytometry analysis

**Figure 1:**
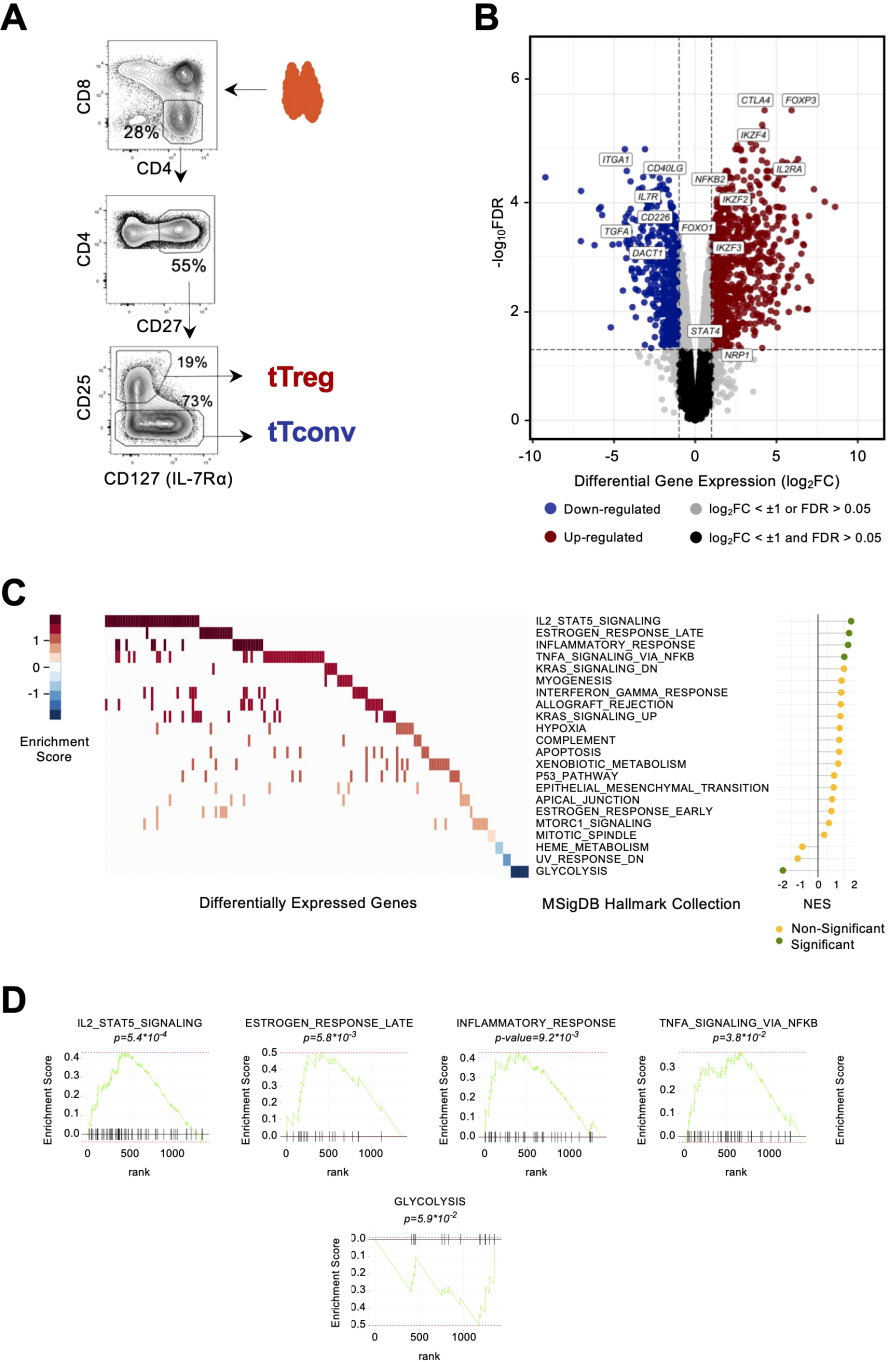
The human thymic Treg signature. **(A)** Illustrative sorting strategy for mature thymic Tregs and their conventional counterparts (tTconv). **(B)** Volcano plot for differentially expressed genes (DEGs, log_2_FC ≥ ± 1, FDR<0.05), representative up (red) and down (blue) DEGs are indicated. **(C)** Heatmap of Gene Set Enrichment Analysis (GSEA) of DEGs, ranked by expression fold-change vs. MSiGDb Hallmark collection; rows, Hallmark signature; columns, DEGs; color scale representing enrichment score, with the lollipop plot showing Normalized Enrichment Score (NES): green, p < 0.05; NES > 0, upregulation; NES < 0, downregulation. **(D)** Enrichment score profiles in the significant GSEA signatures; black bars represent gene matches between the signature and DEGs; rank, position of match in GSEA signature.

Thymocytes isolated by Ficoll-Hypaque (GE Healthcare) from cell suspensions obtained by thymic tissue manual dispersion, were sort-purified to obtain mature CD4 single-positive (CD4SP) regulatory (Tregs) and conventional (Tconvs) thymocytes (purities above 95%), based on the surface expression of CD4, CD8, CD27, CD25, and CD127 using a FACS Aria III (BD Biosciences), as illustrated in **Figure 1A**. CD3 was intentionally not stained to avoid possible signaling, and the sorting strategy was validated in parallel through the analysis of CD3 and intracellular FOXP3 staining in a Fortessa flow cytometer (BD Biosciences), as we previously showed (19). Peripheral mononuclear cells (PBMCs) were isolated from buffy-coats by Ficoll-Hypaque (GE Healthcare); enriched for CD4 T cells by magnetic isolation using the untouched human CD4 T-cell enrichment kit (EasySep, StemCell Technologies); surface stained for TCRαβ, CD4, CCR7, CD45RO, CD25, and CD127; and sort-purified into naïve and memory Tregs and their Tconv counterparts, using a FACS Aria III, as we have previously described (20). Analysis was performed using FlowJo v10 software.

### 2.3 RNA-seq

RNA was extracted from cell pellets of 600,000 sorted thymic, naïve, and memory Tregs and Tconvs from three different thymuses and three different buffy coats, using the AllPrep DNA/RNA kit (QIAGEN) and following the manufacturer’s instructions. A total of 18 libraries were built by selecting for polyadenylated RNA and then sequenced at both ends by high-throughput parallel sequencing (RNA-seq) in an Illumina Hiseq4000 sequencer (BGI Tech Solutions, Hong Kong, China). Raw sequencing was processed and analyzed with SAMtools (21), and sequence quality was assessed with FastQC (see Materials and Tools Table in **Supplementary Material**). The resulting ca. 200 million paired-end reads per biological replicate (PE100) were uniquely mapped and annotated to the human genome (hg38) with TopHat2 (22) and transcript expression was quantified with R package HTSeq (23) (Count Per Million, CPM), with the exclusion of genes with less than 1 CPM in more than two libraries. We controlled for inter-individual variability with multi-dimensional scaling (MDS) analysis, showing that the samples were aggregated by condition irrespective of the donors (**Supplementary Figure 1**). The thymic libraries were used in a previous study (19). Data accession: ArrayExpress E-MTAB-11211 for thymic data and E-MTAB-13930 for peripheral CD4 T cell data.

### 2.4 Differential expression analysis

Libraries were scaled by Trimmed Mean of M-values (TMM) normalization and corrected for heterogeneity of samples specific to contrast matrix with weighted scaling based on voom (24), followed by the quantification of differential expression between Tregs and Tconvs in the thymus and between thymic, naïve, and memory compartments with R package edgeR (25). Finally, we fitted multiple linear models with lmFit. Conversion between annotations was made with R biomaRt (26). False-discovery rate (FDR) corresponds to the adjusted p-value with Holm correction. The cut-off for the expression of 2-fold change warrants the selection for differences with potential biological relevance and was overall on-par with the chosen FDR<0.05. Functional Annotation: Gene Set Enrichment Analyses (GSEA) was performed with fGSEA (https://doi.org/doi:10.18129/B9.bioc.fgsea). The source code for the Gene Set Enrichment Analyses can be found at: https://github.com/AESousaLabIMM/fgsea_msigDB_Thymus_paper. The interactome was generated using the GeneMania plugin for CytoScape 3.10.2, selecting the attributes “Physical interaction”, “Genetic interaction”, and “Pathway”, and spatially arranged using the yFiles Tree layout (27).

### 2.5 Statistical analyses

All quantifications and statistical significance were calculated using R (R Core Team (2023). R: A Language and Environment for Statistical Computing), unless indicated otherwise. All charts and graphs were created with packages from R/Bioconductor. Heatmaps were created with the “ComplexHeatmap” package, volcano plots with the “enhancedVolcano” package, and other charts or graphs with the “ggplot2” package.

## 3 Results

### 3.1 The CD4SP regulatory T cell signature in the human thymus

Genome-wide expression profiles of bulk CD4 single-positive (CD4SP) Treg and Tconv thymocytes (“tTregs” and “tTconvs”) were generated and the human tTreg signature identified as we previously described (19). Briefly, tTregs were isolated from freshly collected thymic tissue based on high CD25 and low CD127 expression levels, and, as tTregs uniformly expressed CD27, CD27+ tTconvs were isolated to control for their maturation status (**Figure 1A**). Bulk RNA-seq of these samples yielded ca. 13,000 genes with non-neglectable expression levels in at least one of the lineages (in E-MTAB-11211), as described in a previous study using these libraries (19). We applied a linear model across all thymic transcriptomes (28) to obtain a high-confidence list of 1,357 DEGS [log_2_(Fold Change) > 1, FDR < 0.05]. The DEG list includes 836 genes with increased expression in tTregs (Up DEGs) and 521 genes more expressed in tTconvs (Down DEGs). Together, they are significantly and sufficiently differentially expressed to define a minimal “human tTreg Expression Signature” (**Figure 1B**, **Supplementary Table 1**).

Consistent with the lineage sorting strategy, the Treg lineage markers *FOXP3*, *CTLA4*, and *IL2RA* (encoding CD25) were highly expressed in tTregs and not in tTconvs, whilst *IL7R* (CD127) was mostly expressed in tTconvs. tTregs also overexpress *IL15RA*, a receptor involved in tTreg development and proliferation (6) and other known markers of Tregs, namely, *DUSP4*; *NR4A3*, the protein of which transactivates *FOXP3* expression (29); and *PRDM1* and *IRF4*, that control differentiation and function of effector Tregs in the periphery (30). Other genes of interest also found to be upregulated in tTregs were those coding for proteins involved in cell trafficking, such as *PERP, CDH1*, and *PCDH12*; cytokine receptors *IFNLR1* and *IL4R*; *RORA*; the chromatin remodeler *HDAC9;* and many currently unreported transcripts potentially required for Treg identity in the human thymus, such as *BCL3* and *IL10RA.* Conversely, Down DEGs included known Tconv genes, such as *CD40LG*; *IL7R*; *ITGA1* (CD49A); *DACT1*; *CD226*, the protein of which competes with immunosuppressive factor TIGIT for the same CD155 ligand; and *TGFA*, which are consistent with reported higher expression in naïve and memory Tconvs compared to corresponding Tregs. We also found several genes known to be involved in Tconv differentiation: *CCR9*, *ITGA1*, *WNT5A*, *CXADR*, and *CEBPB*. Other downregulated genes of interest included *DNM3*, a minus-end oriented microtubule molecular motor*;* integrin *ITGA6*; *FRY*, a mitotic spindle-associated protein; cell motility protein vinculin, *VCL*; *EPAS1*; and *CAMKK1*. Surprisingly, we found the following genes more expressed in Tconvs than Tregs in the human thymus: *RARG*, which binds to the Foxp3-CNS1 to maintain peripheral Tregs (31); and *CAMK4*, with regulatory roles in thymus and periphery (32).

To uncover the key molecular pathways contributing to the human tTreg Signature, we used GSEA and compared our DEG list (ranked by fold-change of expression) with collections of relevant expression signatures. We found a significant hierarchical enrichment in the tTreg Signature (*p* < 0.05) for the pathways “IL2_STAT5_SIGNALING”, “TNFA_SIGNALING_via_NFKB”, “ESTROGEN_RESPONSE_LATE”, “INFLAMMATORY_RESPONSE” (upregulation, **Figures 1C, D**, **Supplementary Table 2**), and “GLYCOLYSIS” (downregulation, **Figures 1C, D**, **Supplementary Table 2**). Amongst the Up DEGs overlapping with the Hallmark sets, we found important signaling molecules such as *IL2RA*, *IL2RB*, *IL10RA*, and *CTLA4* in the case of IL2_STAT5_SIGNALING; or *DUSP4* and *IL15RA*, both in INFLAMMATORY_RESPONSE and TNFA_SIGNALING_via_NFKB. The contribution in enrichment for the latter signature includes POU2F2 (Oct2), the chromatin organizer LMNA, CAV1, and the procadherin PCDH9, in addition to NF-kB pathway members NFKB2, REL, and RELB, the NF-kB pathway inhibitor NFKBIZ, and BHLHE40. Of note, and whilst STAT4 has been described as regulating the IL12 pathway and upstream of Th1 differentiation and cytokines (33, 34), our data showed *STAT4* was more expressed by Tregs than Tconvs in the thymus. Amongst the Down DEGs overlapping with the Halmark “GLYCOLYSIS” was *TGFA*, the important cell-cycle regulator *CDK1*, and genes involved in metabolism, namely *TKTL1*, *NDST3*, and *ALDH7A1*.

The Treg Signature included 56 Transcription Factors (TFs) amongst Up DEGs, with *FOXP3* as the most upregulated (log_2_FC=5.93), and 16 TFs within the Down DEGs. Both groups were overrepresented in the tTreg GSEA signatures previously described (**Supplementary Tables 1**, **2**). These signatures coincide in several upregulated TFs of relevance in the context of T-cell development and differentiation, such as, and in addition to those mentioned above, *IRF8; BCL2A1; NFIA*; a putative pioneer factor, *TGIF1; FOXO1*, which has been implicated in an earlier developmental stage in tTregs; *TGID2*; a group of inducible TFs of the NF-κB pathway, which regulates immune and inflammatory responses and protects cells from undergoing apoptosis in response to cellular stress; and the repressor *RUNX1*, amongst other downregulated TFs in tTregs. Up DEGs included several members of the Ikaros family of TFs, namely, *IKZF2* (Helios), *IKZF3* (Aiolos), and *IKZF4* (Eos), with a well-established role in the Treg lineage definition (35, 36), and the TFs that we have already shown to have a determinant role in the regulation of the thymic Treg signature (19), namely *BATF* from the AP-1 family; *KLF6*, the most expressed member of the KLF family; and *ETV1*, a member of the ETS family.

Altogether, the tTreg Signature confirms genes known to be required for Treg identity and function and identifies many DEGs that merit further exploration. It also provides a unique resource to support research on thymically committed Tregs.

### 3.2 CD4SP Treg “private” genes in the human thymus

**Figure 2:**
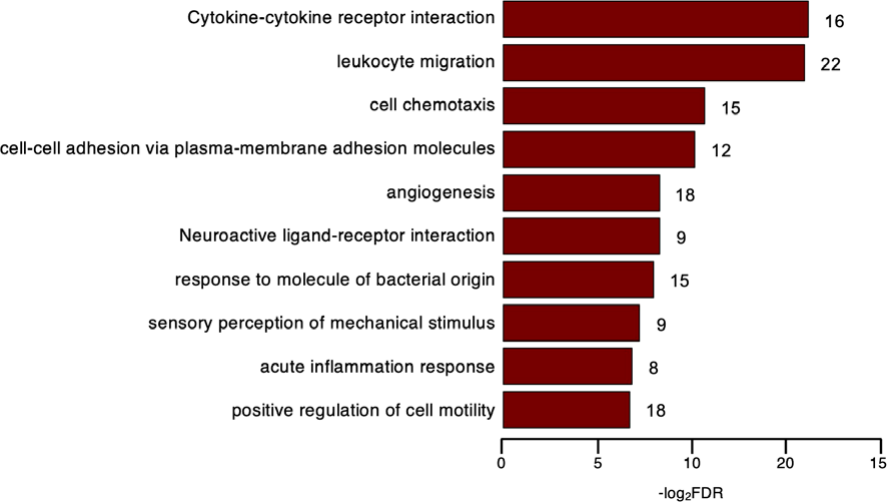
“Private” thymic Treg genes. Over-Representation Analysis (ORA) of the DEGs identified as thymic Treg “private” genes; Capitalized, KEGG Pathway; others, Gene Ontology of Biological Process (GOBP); bar plot, enrichment ratio > 4, FDR < 10^-5^; number of genes found for term in front of bar.

Next, to further explore the tTreg signature, we examined the upregulated genes whose expression was largely ascribed to tTregs. Since tTconvs were sorted based on neglectable CD25 protein levels, we used its coding *IL2RA* transcript levels in tTconvs as a functional threshold to identify 250 DEGs with such distinctive expression in the thymus that we called them tTreg “private” genes (**Supplementary Table 3**). These included several transcripts coding for cytoskeleton, extracellular matrix, and adhesion proteins, such as *ACTG2*, *ACTA2, ACTN2*, *TUBA3E*, *FN1*, the procadherin *PCDH7*, and *ICAM1* (CD54), with functions such as proinflammatory signal transduction in T cells. These genes are involved in cell motility and interact with signaling molecules controlling migration, like Treg marker *NRP1* (CD304) and *RELN* (37). A Gene Ontology (GO) analysis (FDR<0.05, BH correction, Top 10) confirmed the overrepresentation of this function and identified an enrichment for Biological Processes (BPs) related to cell adhesion and migration (**Figure 2**, **Supplementary Table 3**). Included are genes such as the aforementioned *ICAM1*, *FN1*, *PCDH7*, and others, annotated to GO BP terms “cell-cell adhesion via plasma-membrane adhesion molecules”, “acute inflammatory response”, “positive regulation of cell motility”, “sensory perception of mechanical stimulus”, and “acute inflammatory response”.

Moreover, the tTreg “private” genes included chemokines and chemokine receptors, annotated to KEGG and GO BP terms, “Cytokine-cytokine receptor interaction” and “cell chemotaxis”, respectively. In addition to those included in the GO BP terms, we also found *XCL1*, which encodes a chemokine that regulates the establishment of self-tolerance and generation of Tregs in murine thymus (38). It is worth noting that chemokine receptors that have been associated with T-cell effector subsets were included, such as *CXCR3*, associated with Th1; *CXCR5*, a marker of follicular T cells; and *CCR8*, associated with Th2 (**Figure 2**, **Supplementary Table 3**). Consistent with the overexpression of *CXCR3* and *CXCR5*, tTregs express significantly higher levels of *TBX21* and *BCL6*, TFs that are master regulators of Th1 and Tfh differentiation, respectively. Although the peripheral Treg acquisition of polarized profiles that direct their suppressor activity is well-recognized (39), their contribution to the nature and function of Tregs in the human thymus is unknown and subject to speculation. Our results suggest the possibility of commitment to distinct Treg subsets during thymic development, a hypothesis that deserves functional validation in future studies.

Thus, we uncovered two very specialized classes of tTreg “private” genes: one composed of transcript coding for components of the cell movement mechanism, such as extracellular matrix components factors, adhesion, and cytoskeletal proteins; the other comprises specific chemokine and chemokine receptor transcripts, representing proteins linked to cell functional polarization and orchestration of immune subset trafficking and migration.

### 3.3 CD4SP Treg “private” genes with higher expression in thymic than peripheral Tregs

These findings prompted us to investigate whether the tTreg “private” genes are ascribed to the thymus or are comparably expressed after Treg egress in the naïve and memory Tregs.

**Figure 3:**
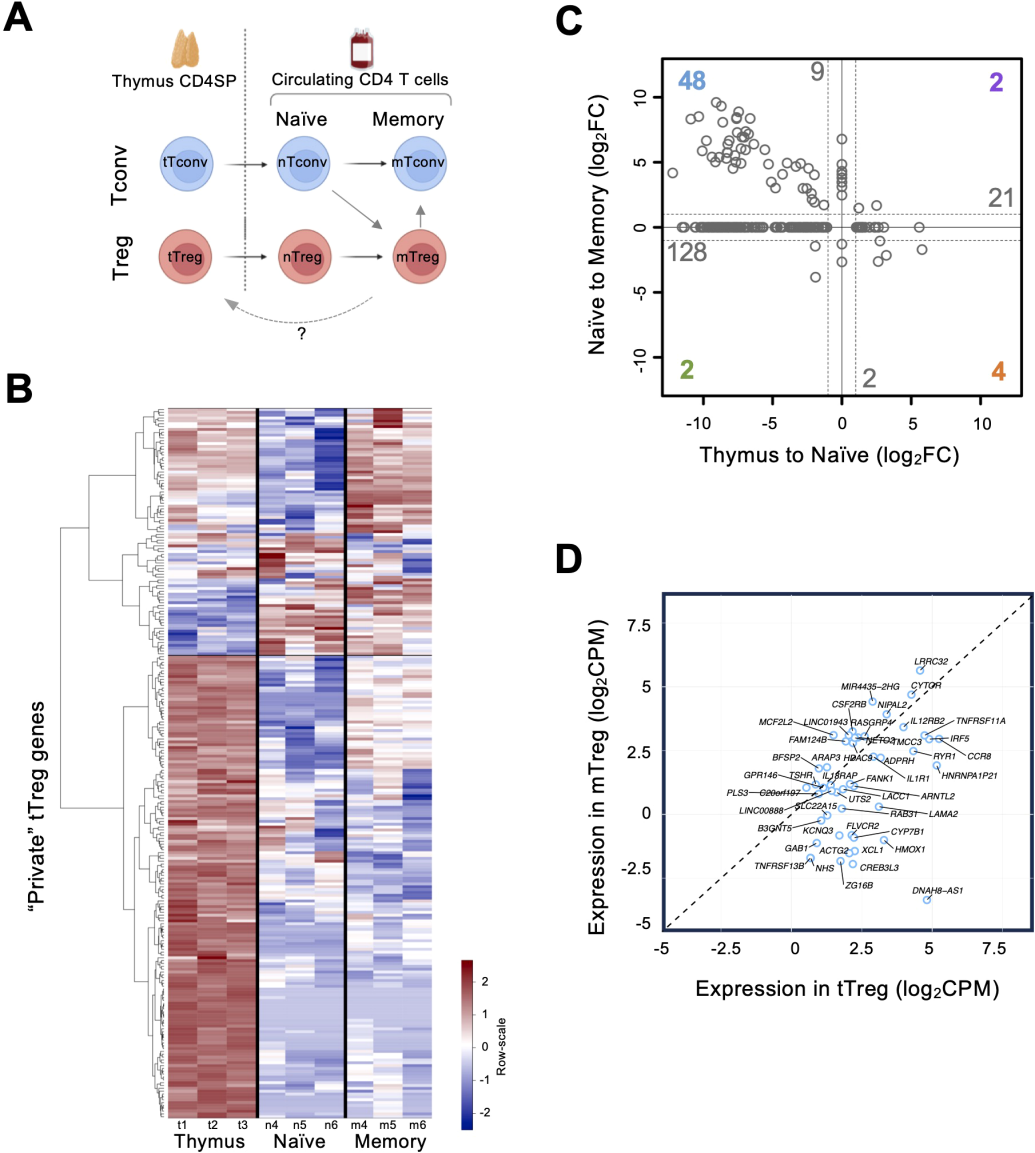
Expression of “private” thymic Treg genes in the peripheral Treg compartments **(A)** Diagram summarizing the study design for sample collection of the two CD4 T-cell lineages (Treg, red, and Tconv, blue), from thymus (t), to naïve (n), and to memory (m); arrows indicate differentiation trajectories, and the dashed arrow illustrates the possibility of mTreg recirculation into the thymus. **(B)** Heatmap of expression for DEGs identified as “private” tTreg genes in the Thymic, Naïve, and Memory compartments (row scale: red, higher; blue, lower). **(C)** Changes in expression (fold-change, FC) for the “private” thymic DEGs between compartments; x-axis, differential expression between thymic Tregs and naïve Tregs (“Thymus to Naïve”, log_2_FC); y-axis, differential expression between naïve Tregs and memory Tregs (“Naïve to Memory”, log_2_FC); numbers refer to the “private” thymic DEGs showing the same changing pattern; color indicates those that changed in the two transitions and gray those that only changed in one; from these, 128 are more expressed in the thymus compared to peripheral compartments. **(D)** Graph showing normalized Counts Per Million (log_2_CPM) in tTregs and mTregs for the 48 “private” genes (blue) more expressed in the thymic and memory compartments than the naïve compartment; 33 DEGs are more expressed in tTregs than in mTregs (below the dashed line).

To test these hypotheses, we generated transcriptomes of corresponding Treg and Tconv subsets in the naïve and memory compartments, as illustrated in **Figure 3A**, harmonized them with the thymic transcriptomes (data availability: E-MTAB-13930), and performed multiple pair-wise comparisons (**Figure 3B**, **Supplementary Table 4**). The analysis included 236 of the above identified tTreg “private” genes with a small discrepancy (14 genes), most likely deriving from the higher stringency in p-values arising from multi-testing (**Supplementary Table 4**).

The hierarchical clustering by expression across compartments revealed different kinetics of expression, the first level clusters segregating a large set of 71% of the tTreg “private” genes more expressed overall in the thymus than in the periphery (**Figure 3B**), with a smaller fraction overexpressed in naïve and memory compartments (**Figure 3B**). Next, we compared the changes of expression between tTregs and naïve Tregs (nTregs), and between nTregs and memory Tregs (mTregs), as shown in **Figure 3C**. This profiling revealed 128 genes that were more expressed in tTregs and were downregulated upon egress into the periphery (**Figure 3C**, **Supplementary Table 5**), which showed a network of high connectivity when annotated to databases of genetic interactions (**Supplementary Figure 2**, **Supplementary Data Sheet 1**). Conversely, 21 genes increased upon egress to the naïve compartment and maintained similar levels in the memory, which included *IL15RA* and *CXCR5*, related to T cell effector functions; *PERP*, involved in p53/apoptotic pathways; and *PCDH12*, *SSH3* and *SELP*, related to cell motility and traffic (**Figure 3C**, **Supplementary Table 5**). The expression levels of 11 genes featured no differences between the thymus and the naïve compartment. Of these, nine (including *ICAM1*, *CXCR3*, and *TBX21* encoding T-bet) increased upon differentiation into memory (**Figure 3C**, **Supplementary Table 5**). Regarding the genes that changed between the two compared transitions, two progressively decreased from thymus to naïve to memory (a pseudogene and *DNAH8* Dynein Axonemal Heavy Chain 8), two increased (*PTGER2* and *OSM*, encoding the receptor 2 of prostaglandin E and oncostatin M, respectively), four were more expressed in naïve but declined upon memory differentiation (*ARG1, TMEM30B, RNF175*, and *CDC14B*, encoding arginase and proteins involved in aminophospholipid transport, ubiquitin pathway, and DNA damage response, respectively), and 48 genes featured lower levels upon thymic egress but increased expression when Tregs differentiate from naïve to memory (**Figure 3C**, **Supplementary Table 5**). We then investigated whether these 48 genes were more expressed in thymic or memory compartments and found that 33 were more expressed in the thymus (**Figure 3D**, **Supplementary Table 6**). Thus, adding these to the previous identified 128, we found 163 tTreg “private” genes to be more expressed in the thymus than in the peripheral compartments (**Supplementary Table 7**), supporting a main role in the thymus, and, conversely, a limited re-use after differentiation into naïve or memory Tregs. **Table 1** lists selected “private” genes, grouped by associated processes relevant to the development of human thymic Tregs. Given the biological processes and pathways associated with the tTreg “private” genes, our data support their role in molecular mechanisms regulating cell chemotaxis/motility and functional specification during Treg development in the human thymus.

**Table 1:** Selected “private” genes with higher expression in thymic than peripheral Tregs.

	HGNC	Name; alias		HGNC	Name; alias
Ion homeostasis	*ACTN2*	Actinin alpha 2	Cell-cell interaction and motility	*ACTG2*	Actin Gamma 2, Smooth Muscle
	*ADPRH*	ADP-Ribosylarginine Hydrolase		*FAT3*	FAT atypical cadherin 3
	*ATP1A4*	ATPase Na+/K+ transporting subunit alpha 4		*FN1*	Fibronectin 1
	*CASQ1*	Calsequestrin 1; calmitine		*ITGB8*	Integrin subunit beta 8
	*ENOX1*	Ecto-NOX disulfide-thiol exchanger 1		*LAMA2*	Laminin subunit alpha 2
	*HAAO*	3-hydroxyanthranilate 3,4-dioxygenase		*NHS*	NHS actin remodeling regulator
	*KCNQ3*	Potassium voltage-gated channel subfamily Q 3		*NRP1*	Neuropilin 1
	*OTOF*	Otoferlin		*PCDH7*	Protocadherin 7
	*PHKA1*	Phosphorylase kinase regulatory subunit alpha 1		*RELN*	Reelin
	*PIEZO2*	Piezo type mechanosensitive ion channel 2		*SLIT1*	Slit guidance ligand 1; *SLIT3*
	*PLS3*	Plastin 3		*TSPAN13*	Tetraspanin 13
	*RYR1*	Ryanodine receptor 1		*TUBA3E*	Tubulin alpha 3e
	*SLC12A8*	Solute carrier family 12 member 8		*CLDN16*	Claudin 16
	*TMPRSS3*	Transmembrane serine protease 3; *TMPRSS4*		*THSD7A*	Thrombospondin type 1 domain containing 7A
	*TMPRSS6*	Transmembrane serine protease 6		*SIGLEC10*	Sialic acid binding Ig like lectin 10
	*CHRNA2*	Cholinergic receptor nicotinic alpha 2 subunit		*CLEC17A*	C-type lectin domain containing 17A
	*FLVCR2*	FLVCR choline and putative heme transporter 2		*FANK1*	Fibronectin type III and ankyrin repeat domains 1
Inflammation	*CCL22*	C-C motif chemokine ligand 22	Inflammation and cell activation	*FGFR3*	Fibroblast growth factor receptor 3; *CD333*
	*CCR8*	C-C motif chemokine receptor 8		*HGF*	Hepatocyte growth factor
	*CX3CR1*	C-X3-C motif chemokine receptor 1		*FST*	Follistatin
	*XCL1*	X-C motif chemokine ligand 1		*HMOX1*	Heme oxygenase 1
	*BTNL8*	Butyrophilin like 8		*PTGS2*	Prostaglandin-endoperoxide synthase 2; *COX2*
	*CLNK*	Cytokine dependent hematopoietic cell linker		*ENPP3*	Ectonucleotide pyrophosphatase/phosphodiesterase 3
	*EBI3*	Epstein-Barr virus induced 3; *IL-27b*		*OXER1*	Oxoeicosanoid receptor 1
	*IL12RB2*	Interleukin 12 receptor subunit beta 2		*A2M*	Alpha-2-macroglobulin
	*IL18RAP*	Interleukin 18 receptor accessory protein		*CNR2*	Cannabinoid receptor 2; *PCDH6*, protocadherin alpha 6
	*IL1R1*	Interleukin 1 receptor type 1		*CAV1*	Caveolin 1
	*IL1RL1*	Interleukin 1 receptor like 1		*ROR1*	Receptor tyrosine kinase like orphan receptor 1; *RORA*
	*IRF5*	Interferon regulatory factor 5		*LYN*	*LYN* proto-oncogene, Src family tyrosine kinase
	*TNFRSF11A*	TNF receptor superfamily member 11a; *RANK*		*ARNTL2*	*BMAL2*, basic helix-loop-helix ARNT like 2
	*TNFRSF13B*	TNF receptor superfamily member 13B; *TACI*		*CREB3L3*	cAMP responsive element binding protein 3 like 3
	*TNFRSF8*	TNF receptor superfamily member 8; *CD30*		*DGCR5*	DiGeorge syndrome critical region gene 5

HGNC, HUGO Gene Nomenclature Committee; HUGO, Human Genome Organization. Alias and processes based on GeneCards: The Human Gene Database annotation.

## 4 Discussion

Bulk RNA sequencing provides a yet-to-be fully explored strategy to decipher cell population identity. This study was carefully designed to sort human CD4SP regulatory and conventional populations matched by their thymic maturation stage based on CD27 expression (1) to produce informative results. Additionally, innovative strategies are required to generate meaningful knowledge from the high-dimensional data generated by NGS (40). Since conventional CD4SPs were sorted based on the lack of CD25 expression, we used the transcriptional level of the corresponding gene, *IL2RA*, to set the threshold for tTreg “private” genes. Additionally, the comparison between circulating naïve and memory Tregs allowed us to generate a unique resource of genes more expressed in tTregs that could inform future studies. This resource will be relevant to improve annotation in single-cell transcriptional studies (41). Moreover, it will help design human studies to validate putative biomarkers for thymically committed Tregs (17).

Our study provided a comprehensive analysis of the expression signature of CD4 Tregs in the human thymus. In addition to the expected main roles in pathways downstream, the TCR and γc-cytokine signaling, our analyses strengthen the role of inflammatory pathways involving possible contributions of IL-1, IL-18, IL-12, and TNFα signaling that has been increasingly recognized (42, 43). We found *TNFRSF1B* encoding the TNF receptor 2, but not *TNFRSF1A* encoding the TNF receptor 1, to be strongly upregulated in tTregs. Future studies should validate how these receptors, known to have distinct physiological outcomes (44–46), determine the downstream impact of this pro-inflammatory cytokine in the tTreg suppressive capacity (47), and how this may be imprinted early in the thymus. As the use of drugs to neutralize TNFs often result in severe side effects, a better understanding of the targeting of TNF receptors in tTregs in chronic inflammation and autoimmune diseases may improve their therapeutic potential. It is also worth emphasizing the negative association with the Halmark “GLYCOLYSIS”, through the enrichment in genes regulating cell cycle and metabolism, in line with studies in peripheral Tregs (48).

Taking advantage of the sequencing depth of our bulk RNAseq approach, we searched for genes expressed in thymic Tregs that are negligible in their Tconv counterparts, which we called “private” thymic Treg genes. As Yayon et al.’s study illustrates (15), most of these genes were not detected by scRNAseq in mature conventional CD4SP datasets from the human thymus (15). Conversely, they were overrepresented in mature CD4SP Tregs (15). Moreover, they were only marginally found in the CD4SP cluster of the “so-called” recirculating Tregs (15), which does not support a contribution of putative recirculating Tregs from the peripheral blood to our thymic data.

Additionally, we identified a set of the tTreg “private” DEGs that were more expressed in the thymic than in circulating Tregs. Thus, although it could not be excluded, our careful comparison with peripheral Tregs is against a contribution of putative recirculating Tregs in our findings. Experimental approaches mapping the cell fate in *in-vitro* cultures may help clarify the origin and the cell differentiation trajectories.

Many of the identified genes are modulators of the cytoskeleton activity and likely play a role in cell motility. Amongst these, there are genes involved in the regulation of ion exchange involving calcium, sodium, and potassium channels, calling attention to the role of these pathways in fine-tuning cell motility and TCR thresholds, as recently reviewed (49). Genes encoding chemokines and chemokine receptors were also identified. Altogether, these results point to the relevance of cell traffic in Treg development and possibly on Treg function, as has been discussed (2, 15, 16). The TCR threshold is the main determinant of agonistic-like Treg selection, promoting the survival of recently committed Treg in the thymus (1). Cell traffic within the thymus is critical for lineage commitment and maturation (1). Cells are thought to migrate rapidly from the cortex into the medulla upon Treg commitment (1, 5, 15, 16), in agreement with our previous modeling of precursor Tregs and their progeny in the human thymus (5). We also found “private” tTreg genes known to be involved in inflammation, allowing us to speculate that these pathways contribute to Treg commitment in the thymus or have an impact on their recognized reduced plasticity (50). It would be relevant to compare the levels of the expression of these genes along Treg development. However, the low frequency of double-positive (DP) Tregs precluded us from doing this analysis using bulk RNAseq. Additionally, there are currently no good strategies to sort DP Tregs since they express high levels of CD127, and FOXP3 cannot be used because it is an intracellular staining.

Our findings are also in agreement with a possible “poised subsetting” of human tTregs, due to the increased expression of chemokine receptors and TFs associated with CD4 T-cell effector functions, as described in peripheral Tregs (39, 50). It is tempting to speculate that enhanced TCR signaling during T-cell development and “premature” activation of *TBX21* and *BCL6* enhancer sites may lead to early upregulation of TFs and chemokine receptors in tTregs and impose imprinting with functional implications (50). This interesting hypothesis merits future experimental validation and characterization of developmental trajectories by flow cytometry and single-cell NGS.

Our study is hindered by the need to compare pediatric thymic samples collected during reconstructive cardiac surgery with samples from the peripheral blood of young adults. This was imposed to reach the cell number required to perform bulk RNAseq that could not be achieved using children’s peripheral blood. Moreover, the experimental confirmation of the individual role of the identified molecules in the human thymus is challenging. Although functional validation studies go beyond the scope of this study, our data are informative for gene network and pathway analyses in future experiments.

Altogether, our transcriptional data represent an important resource to promote the generation of knowledge on human regulatory T cells and T-cell development in the human thymus.

## Data Availability

The datasets presented in this study can be found in online repositories. The names of the repository/repositories and accession number(s) can be found below: https://www.ebi.ac.uk/arrayexpress/E-MTAB-11211 and https://www.ebi.ac.uk/arrayexpress/E-MTAB-13930.
