# Supplementary material for: The distinctive signature of regulatory CD4 T cells committed in the human thymus
Source: Front Immunol. 2025 Mar 26;16:1553554. doi: 10.3389/fimmu.2025.1553554 (PMC11979120; doi:10.3389/fimmu.2025.1553554)
Supplement: Supplementary file 1 [file DataSheet1.pdf]

Supplementary Material

1 Supplementary Data

Supplementary Files

File containing the network in Supplementary Figure 1, Private_genes.cys: https://doi.org/10.5281/zenodo.14575853

2 Supplementary Figure and Tables

2.1 Supplementary Figure

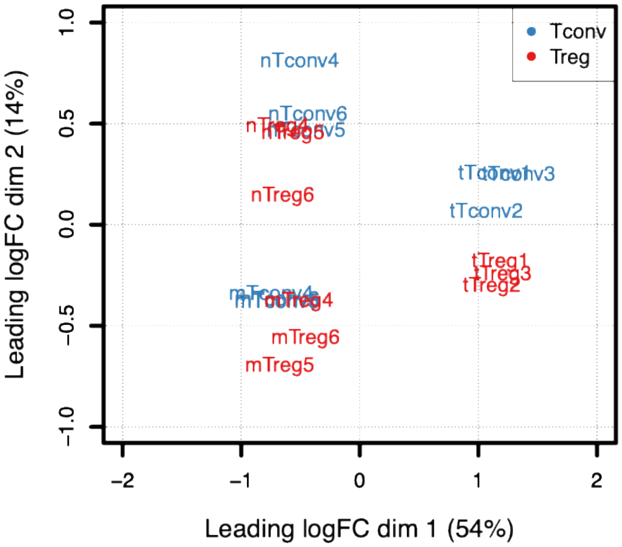

Supplementary Figure 1. ***Multi-Dimensional Scaling analysis of CD4 T-cell transcriptomes.*** Multi-Dimensional Scaling (MDS) of the normalised RNA-seq dataset representing a total of 18 transcriptomes of Tconv (blue) and Treg (red) of the thymic (“t”), naïve (“n”), and memory (“m”) compartments. These correspond to 3 replicates per cell type collected from 6 donors (thymus – 1, 2, 3; peripheral blood – 4, 5, 6). The individual samples aggregate in both dimensions, by their lineage and compartment of origin, and not by donor. (related to Methods).

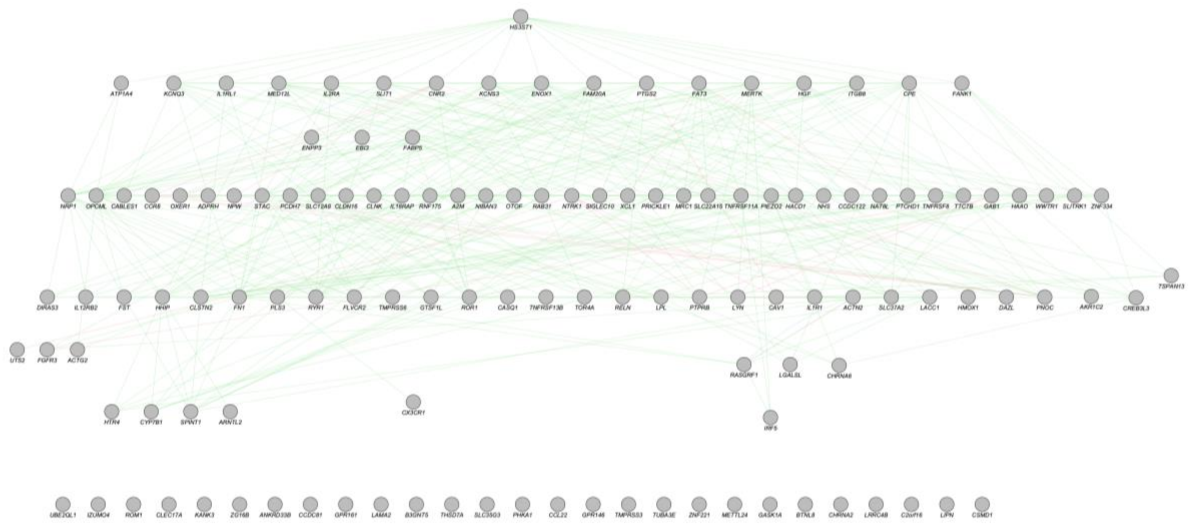

Supplementary Figure 2. ***Interactome of thymic Treg "private" genes more expressed in the thymus than in the periphery and that do not differ in expression in naïve and memory Tregs*** Interactome of 128 DEGs; nodes (black), DEG; edges (undirectional): green, genetic interaction; red, physical interaction; blue, common pathway; design and annotation by GeneMania in CytoScape, see supplementary data Private_genes.cys: https://doi.org/10.5281/zenodo.14575853. (related to Figure 3C, and Supplementary Table 5).

2.2 Supplementary Tables

Supplementary Table 1 ***Human thymic Treg Signature*** (Related to Figure 1B)
(SUPP_TAB_1_FIG1_TregSig.xls)

Supplementary Table 2 ***Gene Set Enrichment analysis of the Human Thymic Treg Signature*** (Related to Figures 1C and 1D)
(SUPP_TAB_2_FIG1C_Hallmark_GSEA_DEGs.xlsx)

Supplementary Table 3 ***"Private" Thymic Treg Genes*** (Related to Figure 2)
(SUPP_TAB_3_FIG2_GO_Private_tTreg_Genes_Thymus.xlsx)

Supplementary Table 4 ***Expression Profile of "Private" Thymic Treg Genes in peripheral Treg compartments*** (Related to Figure 3B)
(SUPP_TAB_4_FIG3B_Private_tTreg_Genes_Periphery.xlsx),

Supplementary Table 5 ***Changes in Expression for "Private" Thymic Treg Genes across compartments*** (Related to Figure 3C)
(SUPP_TAB_5_FIG4A_Private_tTreg_Genes_Compartment_Comparisons.xlsx),

Supplementary Table 6 ***Expression of selected "Private" Thymic Treg Genes in Memory Tregs*** *(*Related to Figure 3D and Supplementary Figure 1)
(SUPP_TAB_6_FIG4B_Private_tTreg_genes_Thymus_vs_Memory_Expression.xlsx)

Supplementary Table 7 ***"Private" Treg Genes more expressed in thymic than in peripheral Tregs*** (Related to Table 1)
(SUPP_TAB_7_TABLE1_Private_tTreg_genes_Thymus_more_than_Periphery.xlsx)
